# Redefining WILD syndrome: a primary lymphatic dysplasia with congenital multisegmental lymphoedema, cutaneous lymphovascular malformation, CD4 lymphopaenia and warts

**DOI:** 10.1136/jmedgenet-2021-107820

**Published:** 2021-12-16

**Authors:** Sahar Mansour, Katherine S Josephs, Pia Ostergaard, Kristiana Gordon, Malou Van Zanten, Julian Pearce, Steve Jeffery, Vaughan Keeley, Katie Riches, Alexander Kreuter, Ulrike Wieland, René Hägerling, Lakshmi Ratnam, Ege Sackey, Dionysios Grigoriadis, Bernard Ho, Frances Smith, Elisabeth Rauter, Peter Mortimer, Derek Macallan

**Affiliations:** 1 Lymphovascular Research Unit, Molecular and Clinical Sciences Research Institute, University of London St George's, London, UK; 2 SW Thames Regional Genetics Service, St George's University Hospitals NHS Foundation Trust, London, UK; 3 Dermatology and Lymphovascular Medicine, St George's University Hospitals NHS Foundation Trust, London, UK; 4 Lymphedema Clinic, Derby Hospitals NHS Foundation Trust, Derby, UK; 5 Department of Dermatology, Venereology and Allergology, Helios St Elisabeth Hospital Oberhausen, University Witten-Herdecke, Oberhausen, Germany; 6 National Reference Center for Papilloma and Polyomaviruses, Institute of Virology, Uniklinik Koln, University of Cologne, Cologne, Germany; 7 Institute of Medical and Human Genetics, Charité Universitätsmedizin Berlin, Berlin, Germany; 8 Radiology Department, St George's University Hospitals NHS Foundation Trust, London, UK; 9 Molecular and Clinical Sciences Research Institute, St George's University of London, London, UK; 10 Viapath Haematology Laboratory, King's College Hospital NHS Foundation Trust, London, UK; 11 Infection Care Group, St George's University Hospitals NHS Foundation Trust, London, UK; 12 Institute for Infection and Immunity, St George's University of London, London, UK

**Keywords:** genetics, medical, haemic and lymphatic diseases, immune system diseases

## Abstract

**Background:**

Primary lymphoedema (PL) syndromes are increasingly recognised as presentations of complex genetic disease, with at least 20 identified causative genes. Recognition of clinical patterns is key to diagnosis, research and therapeutics. The defining criteria for one such clinical syndrome, ‘WILD syndrome’ (**W**arts, **I**mmunodeficiency, **L**ymphoedema and anogenital **D**ysplasia), have previously depended on a single case report.

**Methods and results:**

We present 21 patients (including the first described case) with similar clinical and immunological phenotypes. All had PL affecting multiple segments, with systemic involvement (intestinal lymphangiectasia/pleural or pericardial effusions) in 70% (n=14/20). Most (n=20, 95%) had a distinctive cutaneous lymphovascular malformation on the upper anterior chest wall. Some (n=10, 48%) also had hyperpigmented lesions resembling epidermal naevi (but probably lymphatic in origin). Warts were common (n=17, 81%) and often refractory. In contrast to the previous case report, anogenital dysplasia was uncommon—only found in two further cases (total n=3, 14%). Low CD4 counts and CD4:CD8 ratios typified the syndrome (17 of 19, 89%), but monocyte counts were universally normal, unlike *GATA2* deficiency.

**Conclusion:**

WILD syndrome is a previously unrecognised, underdiagnosed generalised PL syndrome. Based on this case series, we redefine WILD as ‘**W**arts, **I**mmunodeficiency, and**L**ymphatic **D**ysplasia’ and suggest specific diagnostic criteria. The essential criterion is congenital multisegmental PL in a ‘mosaic’ distribution. The major diagnostic features are recurrent warts, cutaneous lymphovascular malformations, systemic involvement (lymphatic dysplasia), genital swelling and CD4 lymphopaenia with normal monocyte counts. The absence of family history suggests a sporadic condition, and the random distribution of swelling implicates mosaic postzygotic mutation as the cause.

## Introduction

In the last decade, there has been significant progress in the classification and understanding of primary lymphatic anomalies and conditions associated with them.^1 2^ Corresponding breakthroughs in gene discovery have revealed at least 20 pivotal genes associated with primary lymphoedema (PL) syndromes.^1–3^ The importance of this progress cannot be underestimated as the identification of responsible molecular pathways opens opportunities to identify new drug targets and potentially life-changing therapies. By contrast, current treatment options for these patients are very limited, relying on compression hosiery, debulking surgery, local therapy for warts and dysplasia, and antibiotic therapy for infections.

Since PL represents a common endpoint of several quite disparate molecular pathways, detailed clinical characterisation is critical for diagnosis and to guide research to improve our understanding of these diseases. Careful evaluation of patient phenotypes and grouping of the patients with similar phenotypes have allowed the definition of specific subtypes of PL and in turn allowed elucidation of their genetic and molecular origin as, for example, with the relationship between lymphoedema distichiasis syndrome (OMIM 153400) and the *FOXC2* gene.^4^ Recognition of new patterns or syndromes is a key step.

**Figure 1:**
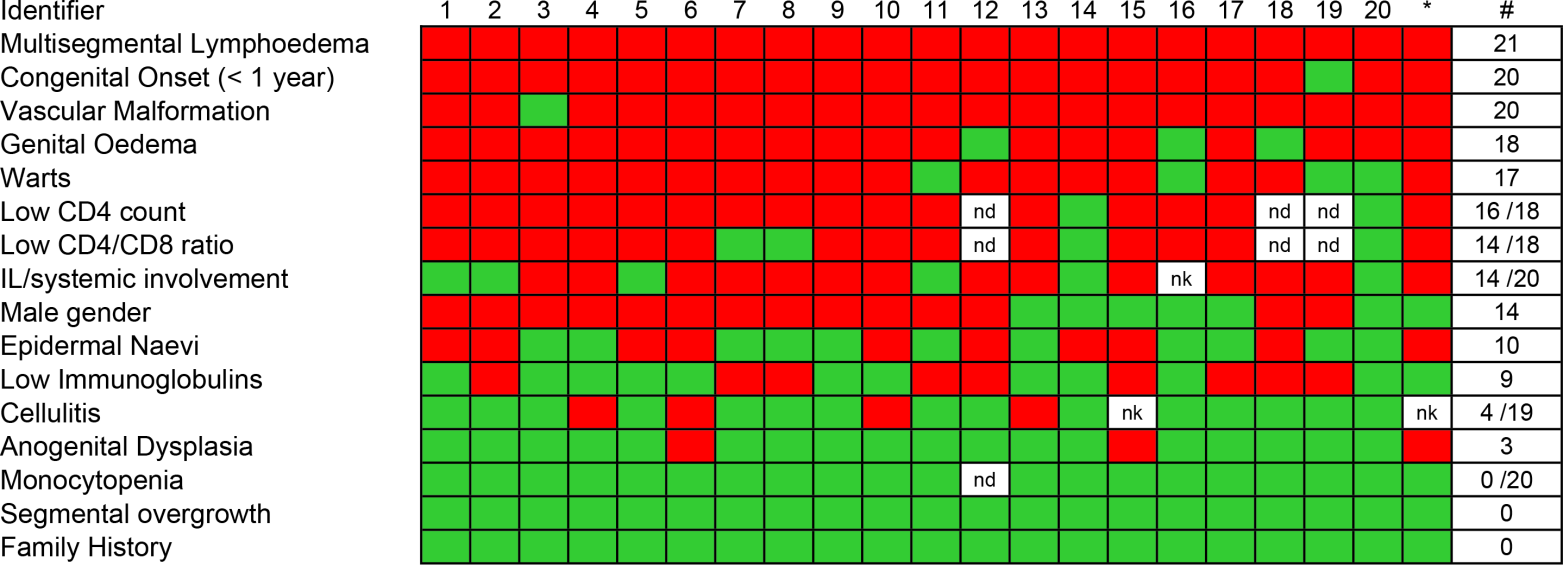
Heatmap of clinical and laboratory features of WILD syndrome ranked in order of frequency. Number of positive cases, total number of cases with available data and frequency are shown on the right. Red indicates present, green is absent and white missing data. *Original case published by Kreuter *et al*.^5^ IL, intestinal lymphangiectasia; ND, not done; NK, not known; WILD, warts, immunodeficiency and lymphatic dysplasia.

In 2008 Kreuter and colleagues^5^ reported a case they termed ‘WILD syndrome’ an acronym for warts, immunodeficiency, lymphoedema and (anogenital) dysplasia. Their patient was a 37-year-old woman with disseminated skin warts from adolescence mainly due to Human Papillomavirus type 57(HPV57); immunodeficiency with profound CD4 lymphopaenia and a reversed CD4:CD8 ratio; PL first noted at 6 months of age in both lower limbs, but later progressing to involve the groin, vulva, anal region and one distal upper extremity; and anogenital dysplasia. In another publication by Yesmin *et al* 6 in 2019, a 13-year-old boy was described with features consistent with WILD syndrome, including warts, impaired cell-mediated immunity, primary multisegmental lymphoedema from birth with systemic involvement and anogenital dysplasia. In the most recent update of the ‘St George’s classification algorithm of primary lymphatic anomalies’,^2^ WILD syndrome was included in the subcategory of ‘multisegmental lymphatic dysplasia’ (‘pink’ section in the original figure 1), a primary lymphatic dysplasia associated with systemic involvement.

We have recently characterised the clinical and laboratory features of a group of patients with congenital multisegmental primary lymphatic dysplasia (defined as any form of lymphatic maldevelopment including lymphoedema and internal lymphatic abnormalities) associated with disseminated warts, and immunodeficiency. We consider that these patients represent exemplars of WILD syndrome as they closely resemble the case previously reported. However, unlike the original report which focused predominantly on comprehensive HPV analyses and excluded epidermodysplasia verruciformis (EV), we now consider ‘WILD syndrome’ as primarily a disease of the lymphatic system in which anogenital dysplasia seems to be less common. The characteristic phenotype is multisegmental lymphoedema with systemic involvement (a lymphatic dysplasia), with a distinctive cutaneous lymphovascular malformation on the anterior chest, and systemic immunodeficiency but not monocytopaenia.

It is important to distinguish WILD syndrome from other syndromes; patients with overlapping symptoms with WILD syndrome have had these attributed to other causes, such as *GATA2* deficiency (OMIM 614038)^7^ or an EV-like condition (OMIM 226400).^8^ However WILD syndrome appears to have distinctive characteristics that make it a recognisable entity. Here, we describe 21 patients with WILD syndrome (20 new cases plus the original case), highlighting the common, consistent features and those that set it apart from other PL syndromes. From these descriptions, we propose novel diagnostic criteria which will not only aid the recognition and diagnosis of WILD syndrome in other patients but will also facilitate ongoing work to discover the molecular cause of this condition.

## Methods

Newly recognised cases (n=20) were recruited from two National Primary Lymphoedema registers in the UK (London and Derby) by systematic clinical phenotyping of all referrals. Written informed consent was obtained from all participants. Laboratory investigations, including genetic testing (see online supplemental methods), and imaging, including lymphoscintigraphy, were performed as clinically indicated. Routinely collected clinical data were retrospectively evaluated with ethical approval. Combining these cases with the previously published case from Kreuter *et al*,^5^ we compiled a cohort of 21 cases which we analysed for common shared clinical and laboratory features.

10.1136/jmedgenet-2021-107820.supp1 Supplementary data

In terms of specific investigations, histology was performed on skin biopsies from two patients. One was from a cutaneous lymphovascular malformation on the left side of the chest and the other from the affected site of the left upper thigh (see online supplemental methods). Lymphoscintigraphy was performed in 17 patients. Radioactive isotope (technitium^99^) was injected into the toe/finger web spaces and images taken at 15 min and 2 hours. The percentage of radioactive isotope remaining in the hand/foot and the percentage taken up by the axillary/inguinal lymph nodes were measured at 2 hours and the rates of retention and uptake were calculated as described previously.^9^ Comparisons between affected and unaffected limbs (excluding patients with bilateral swelling) were made using a paired Student’s t-test.

## Results

The 21 cases comprised 14 male and 7 female patients with median age of 29 (range 11–61). Most, but not all (15 of 21), were white Caucasian. All but one patient reported swelling that began at birth or within the first year of life. None reported a significant family history of similar problems. Commonly shared clinical features are summarised in figure 1 in order of frequency of occurrence.

### Congenital multisegmental lymphoedema

**Figure 2:**
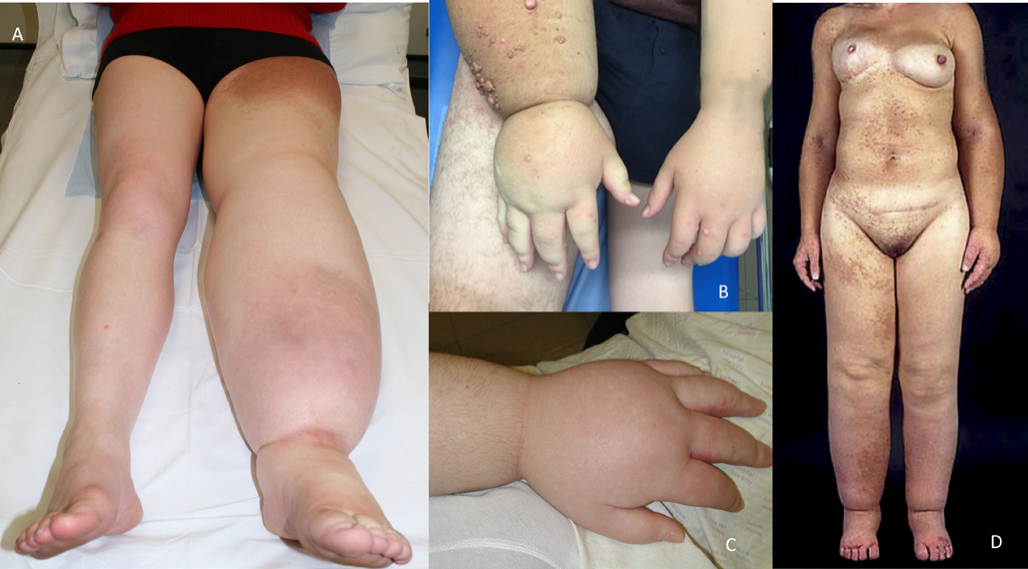
Lymphoedema of the extremities demonstrating unilateral lower limb lymphoedema (A), ‘boxing glove’ hand (B and C) and the ‘multisegmental‘ (mosaic) distribution of swelling of the patient described by Kreuter *et al* 5 (D).

**Figure 3:**
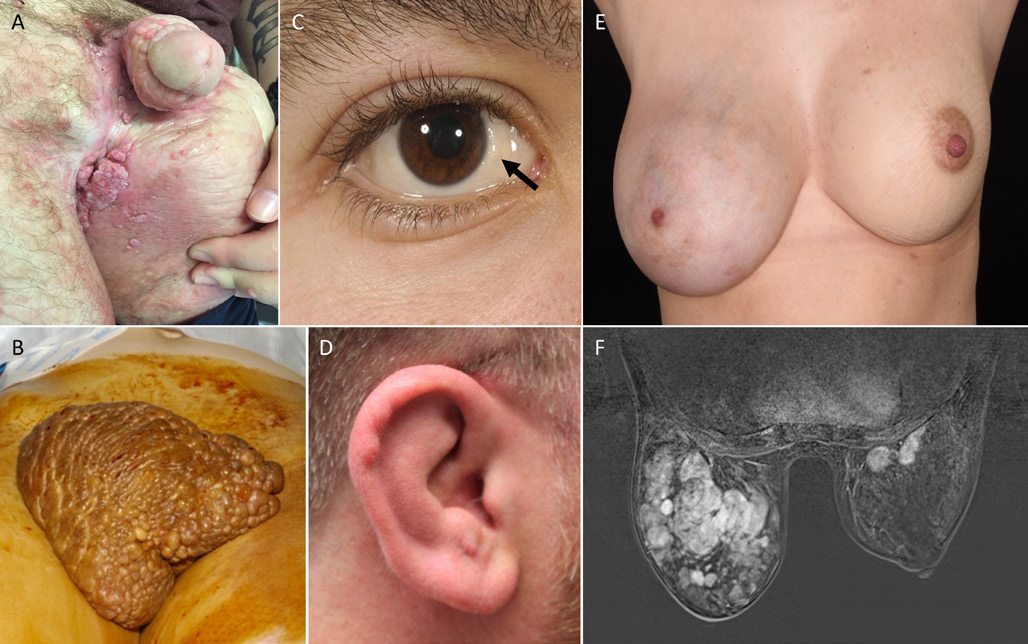
Male genital oedema (A), marked labial oedema (B), conjunctival oedema (C, arrow) and ear oedema (D). Enlarged right breast due to multiple giant fibromas (E), also seen on MRI (F).

The defining feature in this cohort was PL; all 21 (100%) patients had lymphoedema affecting multiple segments. In the majority of cases this affected one or both lower limbs (19 of 21, 90%), one or both upper limbs (19 of 21, 90%) (figure 2), the genital region (18 of 21, 86%; 12 male, 6 female) (figure 3A, B) and one side of the face (15 of 21, 71%), often with conjunctival oedema (4 of 21, 19%) (figure 3C) and sometimes swelling of the helix of the ear (7 of 21, 33%) (figure 3D). Upper limb swelling was often severe, resembling a ‘boxing glove’ hand (figure 2B, C and online supplemental figure 3A). Genital swelling was frequently progressive and dramatic, requiring reduction surgery. The distribution of limb swelling was random and asymmetrical (‘mosaic’), being evenly split between ipsilateral (9 of 21, 43%) (figure 2D) and contralateral, for example, left arm and right leg or vice versa (12 of 21, 57%).

### Systemic lymphatic involvement

Systemic lymphatic involvement was reported in 14 of 20 patients (70%) (there was no information for 1 patient), including the previously described case who had bilateral pleural effusions and hypoalbuminaemia (A Kreuter, personal communication 2019). Eleven had evidence of a protein-losing enteropathy due to intestinal lymphangiectasia (IL). Six of those with IL also had pleural and/or pericardial effusions (6 of 11, 55%). Additionally, three patients presented with pleural and pericardial effusions without evidence of IL.

### Cutaneous lymphovascular malformations and cutaneous manifestations

**Figure 4:**
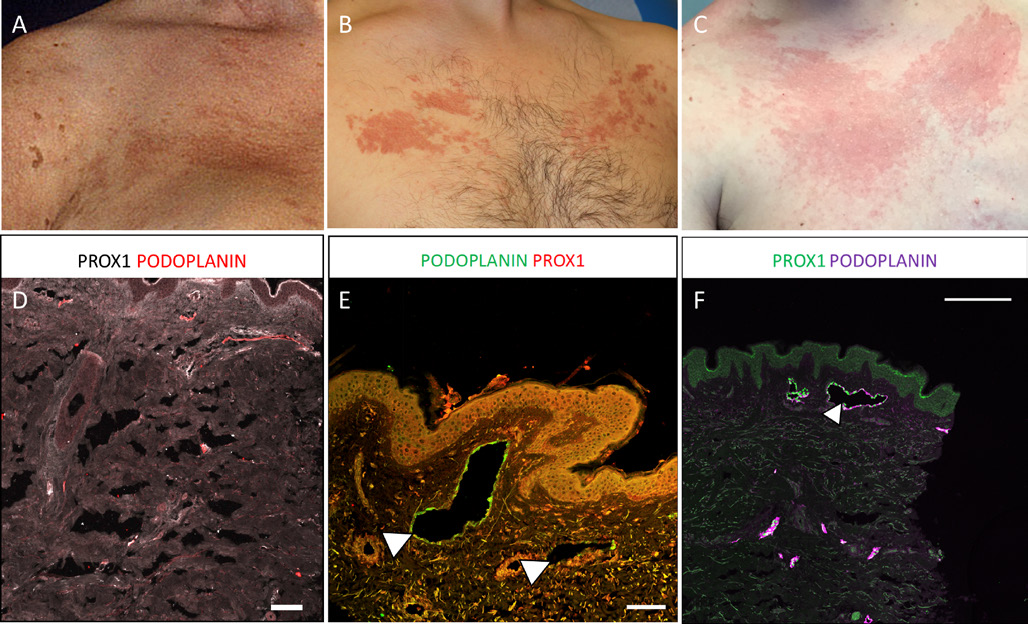
(A–C) Cutaneous lymphovascular malformations predominantly on the upper chest, including the original case described by Kreuter *et al* 5 (A). These were not present at birth but presented during childhood. (D–F) Confocal microscopy with immunohistological detection of lymphatic markers in microtome sections of healthy control (D) and skin biopsies of cutaneous lymphovascular malformations from two patients (E, F) demonstrating dilated dermal lymphatic vessels in patients with WILD compared with control. Detected antigens and respective colours are indicated. Dilated lymphatic vessels are indicated (white arrowheads). Scale bars: 100 µm. Podoplanin, lymphatic-specific endothelial cell surface marker; Prox1, lymphatic-specific endothelial cell nuclei marker; WILD, warts, immunodeficiency and lymphatic dysplasia.

**Figure 5:**
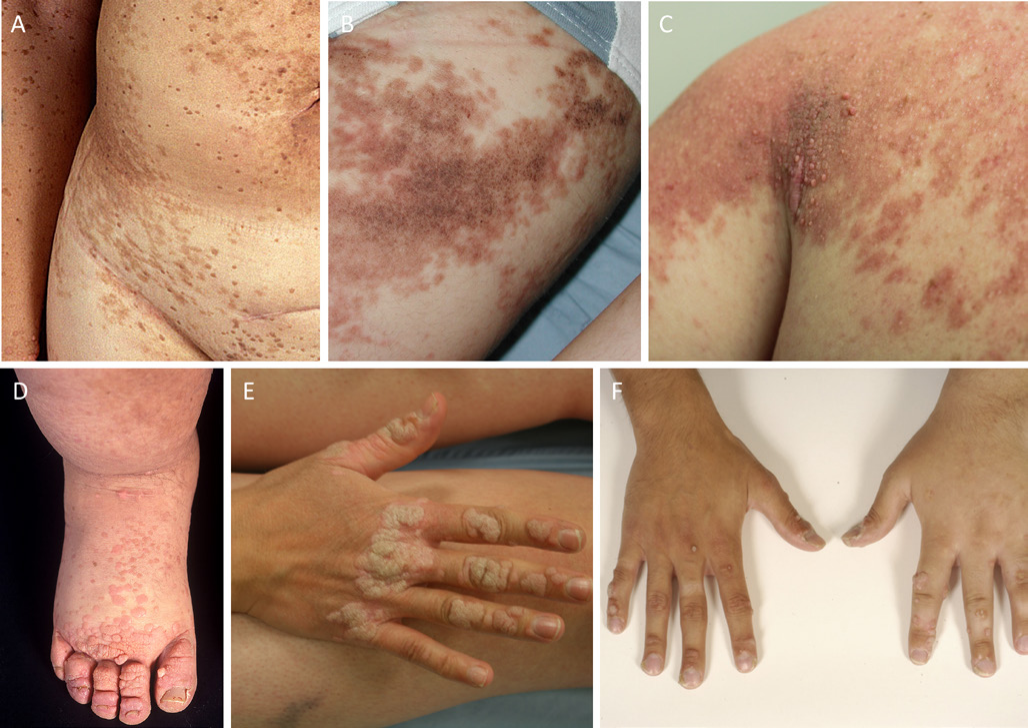
(A–C) Skin lesions suggestive of epidermal naevi. (D–F) Extensive warts of the hand and feet. A and D are photographs of the original patient described by Kreuter *et al*.^5^

Twenty of the patients (95%, including the original case) presented with a cutaneous lymphovascular malformation on either the upper anterior chest wall, neck or thigh (figure 4A–C), the characteristic site being the anterior chest. Histology of two of these lesions identified a lymphatic pathology with dilated lymphatic vessels (figure 4D–F), suggesting these cutaneous malformations may be, at least partly, a lymphatic phenotype. The majority of these cutaneous lymphovascular malformations were not present at birth, typically developing in childhood or early adulthood, and so were not always present at the time of diagnosis. In addition, 10 patients presented with hyperpigmented lesions on the limbs or trunk. Although often described as epidermal naevi, these lesions may represent another manifestation of the underlying lymphovascular malformation, but biopsies were not performed for these lesions, not being clinically indicated (figure 5A–C).

### Immunodeficiency

The most striking immune abnormality seen in these patients was profound CD4 lymphopaenia. Of the 19 with lymphocyte subset data, 17 (89%) had subnormal CD4 counts, with a median of 0.22×10^9^/L (range 0.07–0.99) (figure 1); furthermore, of these 19, 10 were below the 0.20×10^9^/L threshold (online supplemental figure 1). This was reflected by a reduced total lymphocyte count in 14 of 21 (67%) cases. Strikingly, CD8 counts were better preserved, being suppressed in only 7 of 19 assessed (37%), resulting in low CD4:CD8 ratios (<1.56, the lower limit of normal in our laboratory) in all but two subjects (online supplemental figure 1). Importantly, the pattern of immune changes was quite distinct from that seen in *GATA2*-related immunodeficiency, where profound monocytopaenia is characteristic; all subjects (21 of 21) had normal monocyte counts (figure 1). Immunoglobulin levels (IgG, IgA and IgM) were also frequently low (figure 1 and online supplemental figure 2). Although low immunoglobulin levels are a feature of IL-related protein-losing enteropathy, only five of nine patients with low immunoglobulins in this cohort had clinically defined IL.

Despite this apparent cellular and humoral immunodeficiency, clinically evident systemic and/or opportunistic infections were not a notable feature of the syndrome. Local bacterial infections such as recurrent cellulitis in the oedematous limb were reported in 4 of 19 (21 %), but only at rates similar to those seen in other patients with PL syndromes (~25%).^2^ By contrast, viral-driven wart infections were very common in patients with WILD syndrome (see the following section).

### Warts

Of 21 (81%) patients in this cohort, 17 reported current or previous warts (figure 1), and in 7 (41%) of these the warts were severe and persistent despite treatment. While warts do occur in other types of PL, they are typically confined to the swollen limb, presumably due to local immunodeficiency. In this cohort, however, although warts predominantly occurred on the oedematous limb, they also affected other, non-oedematous areas of the body, including the hands and genitalia (figure 5D–F). Genital warts affecting patients with genital lymphoedema were particularly difficult to treat and were often associated with significant discomfort. Only three patients (14%) (including the one original case) demonstrated anogenital dysplasia.

### Other phenotypic features

Segmental overgrowth with limb length discrepancy was not described in this group of patients. The swollen limbs were larger in girth with some evidence of fat hypertrophy (online supplemental figure 3B, C), but there was no limb length discrepancy. Intelligence was normal and these patients had no consistent facial dysmorphic features.

There were several other individual findings: one patient died as a result of a dissected thoracic aorta (cause unknown—aortopathy gene panel was negative); one patient had a left middle cerebral artery infarct with a right hemiparesis secondary to a left internal carotid dissection at the age of 4 years. One patient presented with progressive kyphoscoliosis; one patient was diagnosed with Crohn’s disease and viral myocarditis and another had a diagnosis of coeliac disease. One female patient had several giant fibroadenomas of the breasts which required excision (figure 3E–F); interestingly, the original case had something similar (fibrocystic mastopathy; AK, personal communication). Another female patient suffered from headaches and was diagnosed with benign intracranial hypertension.

### Imaging: lymphoscintigraphy and abdominal imaging

**Figure 6:**
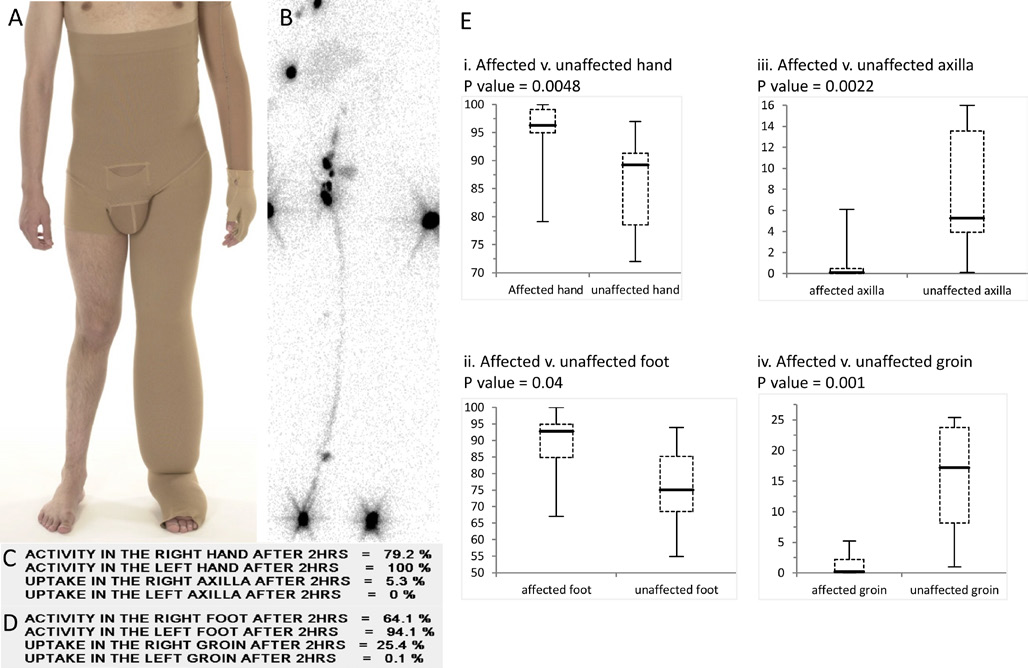
Lymphoscintigraphy of patients with WILD. (A) A patient with multisegmental lymphoedema of the left arm and left leg with compression garments. (B) Four-limb lymphoscintigraphy at 2 hours of the same patient demonstrating unilateral functional aplasia. A and B have been aligned so that injection sites in the feet are at the bottom of the panel and the axillary lymph node visible at the top aligned with the armpits in A. The injection sites in the hands are the dark areas seen on either side of the groin region. (C) Radioactive activity showing marked retention in the left hand and reduction of uptake to the left axilla at 2 hours. (D) Radioactive activity showing marked retention in the left foot and reduction of uptake to the left groin at 2 hours. (E) Affected and unaffected limbs were compared from lymphoscintigraphic imaging of 17 individuals using a paired Student’s t-test. Rates of technitium^99^ retention in the hand (i) and foot (ii) and rates of uptake in the axillary (iii) and inguinal lymph nodes (iv) were significantly different between affected and unaffected limbs in those with unilateral arm or leg involvement (p<0.05). WILD, warts, immunodeficiency and lymphatic dysplasia.

Lymphoscintigraphy in 17 patients demonstrated little or no uptake in the affected limb suggestive of functional aplasia of the initial lymphatic vessels. Affected and unaffected limbs were compared using a paired Student’s t-test. Rates of technitium^99^ retention in the hand and foot and rates of uptake in the axillary and inguinal lymph nodes were significantly different between affected and unaffected limbs in those with unilateral arm or leg involvement (p<0.05) (figure 6).

Abdominal ultrasound and MRI, performed in patients with a history of abdominal discomfort or chronic diarrhoea, demonstrated thickened bowel wall consistent with a diagnosis of IL in four patients (online supplemental figure 3D–F).

### Genetic testing

Sixteen patients (including the previously described patient) had Sanger sequencing or exome sequencing analysis of the *GATA2* gene. No pathogenic or likely pathogenic variants were identified. Four of the patients had extensive testing on DNA (extracted from lymphocytes) of 15 genes known to be associated with PL (the St George’s lymphoedema gene panel); no pathogenic or likely pathogenic variants were identified. Exome sequencing of DNA isolated from peripheral venous blood was carried out for a further 10 of the patients (including the original case from Kreuter *et al* 5) and no pathogenic variants or likely pathogenic variants were identified in any of the 15 lymphoedema genes or the 5 genes associated with EV (*TMC6*, *TMC8*, *CIB1*, *RHOH* and *IL7*).

Two patients had skin biopsies of affected cutaneous lymphovascular malformations and deep read sequencing failed to identify any variants in the *PIK3CA* gene, related phosphoinositide-3-kinase-protein kinase B/Akt (P13K-PKB/Akt) pathway genes or the RASopathy genes.

### Diagnostic criteria

On the basis of the most common defining features of this case series, we suggest the following criteria for a diagnosis of WILD syndrome (with percentages positive in this study in parentheses).

The following are the essential criteria:

- Presence of early-life-onset (<2 years of age) multisegmental PL (100%).

The following are the major criteria (occurring in at least 70% of cases):

- Cutaneous lymphovascular malformation on the chest or neck (95%) (may not be present in the first few years of life).
- Genital swelling (86%).
- Recurrent warts (81%).
- CD4 lymphopaenia (89%).
- Systemic involvement (70%) (ie, IL, pleural effusions, pericardial effusions).

In the absence of the following:

- Low monocyte count.
- Family history of similar malformation.
- Segmental overgrowth with limb length discrepancy.

Although the previously published case suffered from anogenital dysplasia, it was only a feature in two of our new cases (making a total of three in this case series), so we suggest that it is not a major diagnostic criterion. Similarly, although arteriopathy was observed in two patients (dissection of the thoracic aorta and a middle cerebral artery infarct), being infrequent, we have omitted it from the major diagnostic criteria. WILD should therefore be considered an acronym for warts, immunodeficiency and lymphatic dysplasia syndrome.

Applying our criteria, the presence of the essential criterion plus at least two positive major criteria (out of five) and the absence of the three exclusion criteria would have identified all 21 cases.

## Discussion

In this report, we describe 21 patients with clinical features (figure 1) in keeping with WILD syndrome, which we have redefined as warts, immunodeficiency and lymphatic dysplasia syndrome. This is a newly characterised form of PL for which the main diagnostic features are early-onset (often congenital) multisegmental lymphoedema, often with a distinctive cutaneous lymphovascular malformation on the anterior chest and with internal lymphatic dysplasia (systemic involvement). It is typified by distinct systemic immunodeficiency (CD4 lymphopaenia), HPV-induced warts, and in some cases anogenital dysplasia. In our view, WILD syndrome represents a clinically recognisable condition that is distinct from its two most closely overlapping syndromes: *GATA2* deficiency (Emberger syndrome) and EV.

First, in terms of the distinction from *GATA2* deficiency, although some of the clinical features such as PL and warts are shared, the overall clinical presentation, pattern of cellular abnormalities and occurrence of opportunistic infections are markedly dissimilar between the two syndromes. Unlike patients with WILD syndrome, patients with *GATA2* deficiency rarely have lymphoedema affecting the face or upper limbs (the swelling is usually confined to one or both lower limbs and the genital region) and do not present with cutaneous lymphovascular malformations or epidermal naevi. Furthermore, while *GATA2*-deficient patients have profound monocytopaenia, immunodeficiency and an increased risk of myelodysplasia and acute myeloid leukaemia, none of the patients with WILD in this series had either abnormal monocyte counts or developed myelodysplasia. No pathogenic *GATA2* variants were identified through (Sanger and/or exome) sequencing of 16 of our patients, further supporting the notion that WILD and *GATA2* deficiency are separate conditions. A previous paper suggesting that WILD syndrome was caused by a cryptic intragenic deletion in *GATA2* described a patient with characteristics more akin to *GATA2* deficiency than WILD syndrome, with monocytopaenia (0.01×10^9^/L) and no (mentioned) cutaneous lymphovascular malformations or epidermal naevi.^7^ Similarly, the patient described by Ostrow *et al* 8 with lymphoedema, impaired cell-mediated immunity and Bowen’s disease (malignant transformation of HPV infection of the thumb) also almost certainly had *GATA2* deficiency; the patient’s brother had died of leukaemia, the lymphoedema affected both lower limbs, and vascular malformations and epidermal naevi were not reported.

Second, we contend that WILD syndrome is distinct from EV. EV is characterised by widespread, persistent infection by HPV. Lymphoedema and lymphatic dysplasia are not features of EV. Kreuter *et al* 5 demonstrated that the HPV strains found in their patient were not typical of EV, although in both conditions HPV infection may undergo malignant transformation. A number of different genetic causes of immunodeficiency may give rise to EV, five of which are listed in OMIM, all with autosomal recessive inheritance. No pathogenic variants were identified in these genes in any of the 10 patients who underwent exome sequencing. This clinical and likely genetic distinction between WILD and EV does not, of course, exclude a potential role for HPV in the dermatopathology observed in our patients with WILD.

Immunodeficiency is not uncommon in patients with lymphatic abnormalities.^10^ Infections such as cellulitis and warts due to human papilloma virus are common and often recurrent and resistant to treatment. While there is general agreement that tissue immunodeficiency exists within the region of compromised lymph drainage due to a failure of immune cell trafficking, deficiencies in systemic lymphocyte subsets, in particular reduced CD4 counts and low CD4 to CD8 ratios, are also often noted in patients with lymphoedema (PSM, personal communication). Such features were particularly marked in the WILD syndrome cases described here, where lymphopaenia, and specifically CD4 lymphopaenia, was almost a defining feature: 17 of 19 cases tested had subnormal CD4 counts and 10 had values <0.2×10^9^/L, considered indicative of clinically significant immunodeficiency in other settings. Hypogammaglobulinaemia was also common in patients with WILD. A similar pattern of predominant CD4 lymphopaenia and hypogammaglobulinaemia is well described in patients with IL.^11^ In this series, 11 cases were diagnosed with IL, and the others may have had subclinical undiagnosed IL, but intestinal losses are unlikely to represent the sole explanation for the observed cellular deficiencies, not least because they fail to account for the disparate effects on CD4 and CD8 cells. Other factors likely to operate include local entrapment of lymphocytes in the lymphoedematous limb, upregulation of apoptosis induced by increased Fas (CD95) levels, as described in IL,^12^ disordered lymphopoiesis/thymic atrophy,^13^ and disordered interactions between the lymphoid cells and the lymphatic endothelial cells.^10^ The interaction between systemic immunodeficiency and lymphoedema is likely to be bidirectional and complex. For example, lymphoedema is also seen in some cases of Interleukin 2 Receptor Subunit Gamma (IL2RG)/Janus Kinase 3 (JAK3) severe combined immunodeficiency, in which a primary lymphopoietic disorder appears to modify lymphangiogenesis.^14^

Despite these abnormalities, apart from HPV infection, opportunistic or systemic infections were not frequently noted. This pattern resembles that seen in WHIM syndrome (warts, hypogammaglobulinaemia, infections and myelokathexis; OMIM 193670), where susceptibility to HPV appears disproportionate to the overall susceptibility to other infections.^15^ Pathophysiological events likely follow a sequence in which immunodeficiency, particularly affecting T cell function, results in uncontrolled local HPV infection, resulting in severe and persistent warts. In some individuals, specific oncogenic HPV genotypes then drive neoplastic transformation, resulting in anogenital dysplasia. The infrequency of anogenital dysplasia in our cohort (3 of 21) may relate to their relative youth (median age 29), placing them at an earlier stage in this pathological sequence. With long-term follow-up, more cases may develop. HPV typing would have been informative in the cohort described here but is not readily available. This will be the focus of future studies. Anogenital HPV prevalence also depends on sexual activity and HPV vaccination status. Early diagnosis and focused management within specialist centres may reduce the risk of developing anogenital dysplasia.

In this report, none of the patients described a family history of similar symptoms or related problems. This suggests that WILD syndrome is a sporadic condition, the underlying molecular cause of which is yet to be determined. In view of the marked asymmetry in the distribution of the swelling, we hypothesise that this condition most likely arises from a mosaic postzygotic mutation. If so, a germline mutation in the causative gene may well be embryonically lethal. We have looked for *PIK3CA* and related gene mutations in the AKT pathway and the RASopathy genes in DNA from affected skin in two of our patients. No mutations were identified. Unlike patients with *PIK3CA* or RASopathy postzygotic mutations, our patients with WILD syndrome do not have segmental overgrowth or arteriovenous malformation. It is rapidly becoming established that many sporadic forms of vascular malformations (including lymphatic malformations) are caused by postzygotic (somatic) mutations affecting the malformed tissue alone.^16^ In these patients, we would not expect to see a family history of similar problems. In addition, there can be variation in the location and severity of symptoms depending on the timing of the mutation during development. The advent of next-generation sequencing technologies used for deep sequencing of affected tissue has vastly improved the detection rate of low-level somatic mutations which may aid this process. Indeed, the medical literature is now expanding with the discovery of mosaic postzygotic mutations causing isolated or segmental defects, not only in the context of vascular malformations, but also in other diseases.^17^

Determining the genetic cause of PL syndromes and vascular malformations opens the door to discovery of new targets for drug therapy. Mammalian target of rapamycin **(**mTOR) inhibitors, immunosuppressants used in transplant medicine, have been repurposed to target patients with *PIK3CA*-related vascular malformations and segmental overgrowth. Results from a recent clinical trial have shown that low-dose sirolimus (an mTOR inhibitor) can significantly reduce overgrowth of affected areas.^18^ Drugs known to target the Mitogen-activated protein kinase (MAPK)/extracellular signal-regulated kinases (ERK) pathway (also known as the Ras-Raf-MEK-ERK pathway) pathway are already available and being used in cancer therapy.^19^ These could represent a new treatment for selected patients with mosaic RASopathies.^16^

In summary, WILD syndrome is a clinically recognisable condition characterised by congenital multisegmental lymphoedema (often asymmetrically distributed and usually of early onset) with systemic involvement, cutaneous lymphovascular malformations, severe and persistent warts and epidermal naevi in association with CD4 lymphopaenia (and consequently low CD4:CD8 ratios), but normal monocyte counts. The cutaneous lymphovascular malformations may not be present until later in childhood. Their exact nature has not been fully explored and the lack of histological information is a limitation of this study, but in the two cases that were biopsied a lymphatic pathology with dilated lymphatic vessels was identified, suggesting at least a lymphatic component. It is likely that these are poorly differentiated vessels with lymphatic and vascular components. This is also a focus of further research in our group. The lack of family history suggests it is a sporadic, mosaic condition possibly due to a postzygotic mutation.

Our conclusions are based on data from 21 patients so may be subject to sampling error; as more cases are defined, the true prevalence of each feature will become clearer and the definition may need to be revisited. However, on the basis of current evidence, we provide a case definition comprising the presence of the essential criterion (early-onset multisegmental lymphoedema), plus at least two of the five major criteria, and the absence of three exclusion criteria. We envisage that the clinical information and diagnostic criteria presented here will not only aid the diagnosis of other patients with WILD syndrome but will also assist with work uncovering the molecular cause. Deep sequencing and sophisticated analysis of affected tissue from patients will be needed to investigate whether a novel gene or molecular pathway is responsible for this condition.

## Data Availability

All data relevant to the study are included in the article or uploaded as supplementary information.
