# Supplementary material for: Redefining WILD syndrome: a primary lymphatic dysplasia with congenital multisegmental lymphoedema, cutaneous lymphovascular malformation, CD4 lymphopaenia and warts
Source: J Med Genet. 2021 Dec 16;60(1):84–90. doi: 10.1136/jmedgenet-2021-107820 (PMC9811088; doi:10.1136/jmedgenet-2021-107820)
Supplement: Supplementary data [file jmedgenet-2021-107820supp001.pdf]

Supplementary Material

Redefining WILD syndrome: A primary lymphatic dysplasia with congenital multisegmental lymphoedema, cutaneous vascular malformation, CD4 lymphopenia and warts

Sahar Mansour[1,2], Katherine Josephs[1,2], Pia Ostergaard[1], Kristiana Gordon[3], Malou Van Zanten[1], Julian Pearce[3], Steve Jeffery[1], Vaughan Keeley[5], Katie Riches[5], Alexander Kreuter[6], Ulrike Wieland[7], René Hägerling[8], Lakshmi Ratnam[1,4], Ege Sackey[1], Dionysios Grigoriadis[1], Bernard Ho[3], Frances Smith[9], Elisabeth Rauter [9], Peter Mortimer[1,3], Derek Macallan[1,10,11].

Supplementary Methods

Genetic Testing

***GATA2* screening.** Sanger sequencing of the *GATA2* gene was carried out on DNA extracted from blood lymphocytes at St. George's for 13 of the affected individuals. The coding regions and associated splice sites of *GATA2* were Sanger sequenced from six PCR amplicons. Primers were designed using Primer3 software (Rosen et al., 2000). PCR products were purified with ExoSAP-IT (GE Healthcare) and sequenced using BigDye Terminator v3.1 chemistry (Life Technologies) on a 3730 DNA analyser (Life Technologies). Sequence traces were aligned to reference using SeqScape software (v2.0) (Life Technologies).

Sequencing of skin biopsies. DNA was extracted from skin fibroblasts from affected sites and screened for mutations in the PIK3CA gene-related AKT pathway genes or the RASopathy genes by deep-resolution, next generation sequencing in an NHS accredited lab.

Lymphoedema panel. DNA from blood was screened for pathogenic variants in 15 genes (*ADAMTS3, CCBE1, EPHB4, FAT4, FOXC2, GATA2, GJA1, GJC2, GJC2, KIF11, PIEZO1, PTPN14, SOX18, VEGFC, VEGFR3*) associated with lymphoedema using an NHS accredited laboratory.

Exome sequencing. Sequencing libraries were made following the protocol from Roche/Nimblegen's SeqCap EZ Exome Library v2.0 kit. The libraries were then sequenced on HiSeq2000 (Illumina) machines. Sequence reads were aligned to the reference genome (hg19) using Novoalign (Novocraft

Technologies). Duplicate reads, resulting from PCR clonality or optical duplicates, and reads mapping to multiple locations were excluded from downstream analysis. Depth and breadth of sequence coverage were calculated with custom scripts and the BedTools package (Quinlan et al., 2010). All variants were annotated using a custom annotation pipeline. Single-nucleotide substitutions and small indel variants were identified and quality filtered within the SamTools software package (Li et al., 2009) and in-house software tools (Simpson et al., 2011). Variants were annotated with respect to genes and transcripts with the Annovar tool (Wang et al., 2010).

Processing of skin biopsies for confocal microscopy with immunohistological detection of lymphatic markers

Skin biopsies were fixed for 4 hours in 4% PFA/PBS, washed in PBS, embedded and snap-frozen in OCT. 10 µm cryosections were, washed and blocked (10% chicken serum, 0.3% Triton X-100 in PBS). Subsequently, sections were incubated for 1 hour with primary antibodies (diluted in 1% BSA, 1% chicken serum, 0.3% Triton X-100 in PBS), washed three times in PBS-T and finally incubated in Alexa dye–conjugated secondary antibodies (Life Technologies). After sample mounting in Mowiol (Calbiochem, 475904), confocal images were captured using a Zeiss LSM 700 (×20, NA = 0.8) confocal microscope. The following antibodies were used: rabbit polyclonal anti-human PROX1 (102-PA32, Reli-aTech), mouse anti-human PDPN (clone D2-40, Dako).

Supplementary Figures: Supplementary Figure 1. Lymphocyte profiles in WILD patients

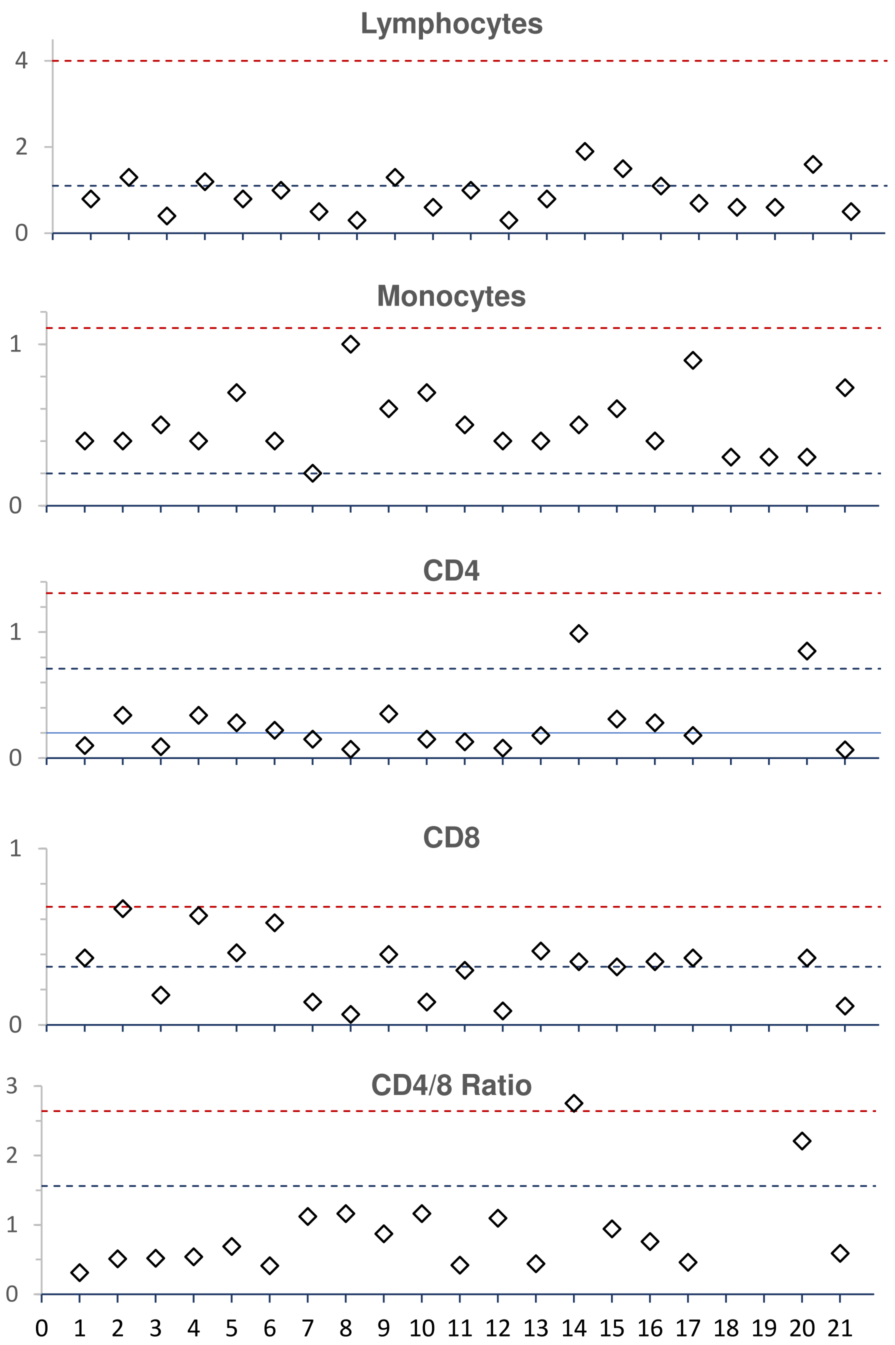

Values on y-axis represent individual lymphocyte subset counts (x10^9 cells/L, except for CD4/8 which is a ratio); numbers are subject identifiers. Red dotted line, upper limit of normal; blue dotted line, lower limit of normal; solid blue line, 0.2 x10^9 cells/L threshold. Subset data missing for subject #18 and 19.

Supplementary Figure 2. Immunoglobulin levels in WILD patients

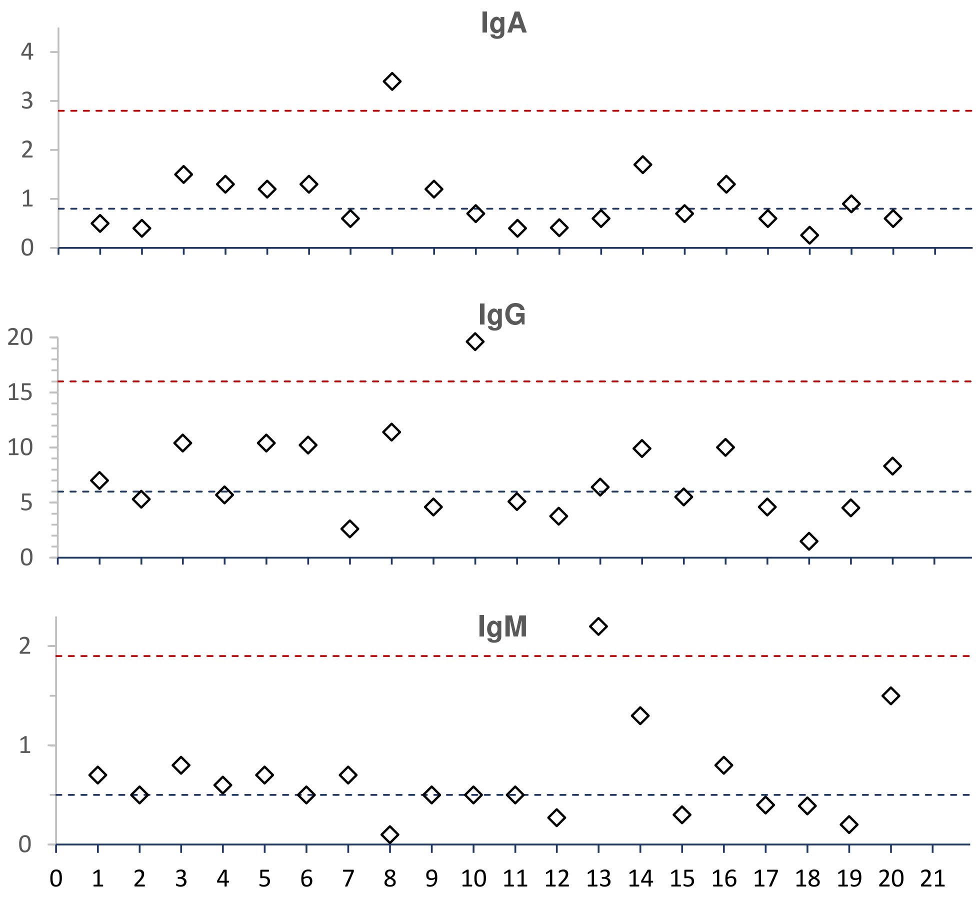

Values on the y-axis represent individual serum immunoglobulin levels in g/L; numbers are subject identifiers. Red dotted line, upper limit of normal; blue dotted line, lower limit of normal. Two low serum immunoglobulin levels out of three leads to a "low immunoglobulins" score in Table 1. No data available for subject #21.

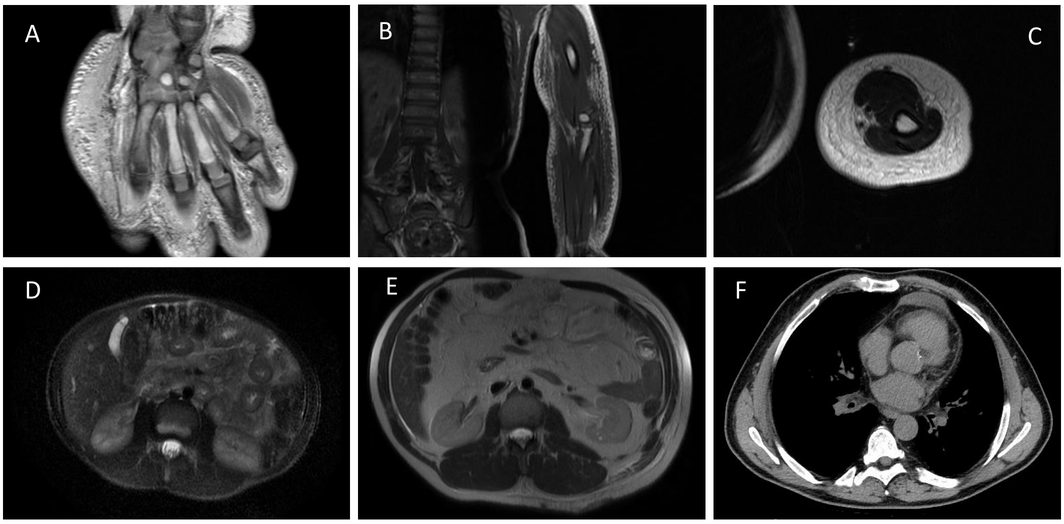

Supplementary Figure 3. MR imaging in WILD patients. MR imaging demonstrating 'boxing glove' lymphoedema of the hand (A), lymphoedema of the arm (B, sagittal; C, coronal). Jejunal wall thickening (D), mesenteric lipohypertrophy (E) and pericardial effusion (F).

Supplementary References

Li H, et al. The sequence alignment/map format and SAMtools. Bioinformatics. 2009;25(16):2078–2079.

Quinlan AR, Hall IM. BEDTools: a flexible suite of utilities for comparing genomic features. Bioinformatics. 2010;26(6):841–842.

Rozen S, Skaletsky H. Primer3 on the WWW for general users and for biologist programmers. Methods Mol Biol. 2000;132:365–386.

Simpson MA, et al. Mutations in NOTCH2 cause Hajdu-Cheney syndrome, a disorder of severe and progressive bone loss. Nat Genet. 2011;43(4):303–305.

Wang K, et al. ANNOVAR: functional annotation of genetic variants from high-throughput sequencing data. Nucleic Acids Res. 2010;38(16):e164.
